# Cellular and Exosomal Regulations of Sepsis-Induced Metabolic Alterations

**DOI:** 10.3390/ijms22158295

**Published:** 2021-08-02

**Authors:** Michael G. Appiah, Eun Jeong Park, Yuichi Akama, Yuki Nakamori, Eiji Kawamoto, Arong Gaowa, Motomu Shimaoka

**Affiliations:** 1 Department of Molecular Pathobiology and Cell Adhesion Biology, Mie University Graduate School of Medicine, 2-174 Edobashi, Tsu-City 514-8507, Mie, Japan; 317DS06@m.mie-u.ac.jp (M.G.A.); y-akama@clin.medic.mie-u.ac.jp (Y.A.); 318DS07@m.mie-u.ac.jp (Y.N.); a-2kawamoto@med.mie-u.ac.jp (E.K.); arong-g@doc.medic.mie-u.ac.jp (A.G.); 2 Department of Emergency and Disaster Medicine, Mie University Graduate School of Medicine, 2-174 Edobashi, Tsu-City 514-8507, Mie, Japan

**Keywords:** sepsis, exosomes, metabolism, endothelial cells, immune cells

## Abstract

Sepsis is a sustained systemic inflammatory condition involving multiple organ failures caused by dysregulated immune response to infections. Sepsis induces substantial changes in energy demands at the cellular level leading to metabolic reprogramming in immune cells and stromal cells. Although sepsis-associated organ dysfunction and mortality have been partly attributed to the initial acute hyperinflammation and immunosuppression precipitated by a dysfunction in innate and adaptive immune responses, the late mortality due to metabolic dysfunction and immune paralysis currently represent the major problem in clinics. It is becoming increasingly recognized that intertissue and/or intercellular metabolic crosstalk via endocrine factors modulates maintenance of homeostasis, and pathological events in sepsis and other inflammatory diseases. Exosomes have emerged as a novel means of intercellular communication in the regulation of cellular metabolism, owing to their capacity to transfer bioactive payloads such as proteins, lipids, and nucleic acids to their target cells. Recent evidence demonstrates transfer of intact metabolic intermediates from cancer-associated fibroblasts via exosomes to modify metabolic signaling in recipient cells and promote cancer progression. Here, we review the metabolic regulation of endothelial cells and immune cells in sepsis and highlight the role of exosomes as mediators of cellular metabolic signaling in sepsis.

## 1. Introduction

Sepsis is a life-threatening organ dysfunction caused by dysregulated host response to infection [1]. Approximately 11 million people died of sepsis globally in 2017 [2]. The pathophysiologic feature of organ dysfunction in sepsis is a dysregulated host response that comprises hyperinflammation and immunosuppression (a.k.a., immune paralysis). It is notable that hyperinflammation and immunosuppression coexist from the onset of sepsis [3]. Besides these contrasting immune responses, apoptosis, which is another pathological process of sepsis, is thought to contribute to eliciting immunosuppression and relapsed infections [4,5]. We will briefly discuss on this process during sepsis in Section 2.2.

Over the years several clinical trials of anti-inflammatory therapies such as the use of corticosteroids, activated protein C, tumor necrosis factor receptor Fc (TNFR-Fc) fusion protein, anti-tumor necrosis factor α (anti-TNF-α), thrombomodulin, and anti-interleukin1 receptor antagonist (IL-1ra) have failed to demonstrate the improvement of mortality [6,7,8,9]. With regards to immunomodulatory therapies to reverse immune paralysis, administration of IL-7 and inhibition of programmed cell death protein 1/programmed death ligand (PD-1/PD-L) interaction are promising investigational drugs, although their clinical effectiveness has yet to be shown [10,11]. On the other hand, granulocyte-macrophage colony-stimulating factor (GM-CSF) therapies have shown an effect to improve symptoms of adult patients with severe sepsis or cirrhosis by restoring innate immune responses [12,13] and pediatric patients with multiple organ dysfunction syndrome (MODS) by preventing nosocomial infection [14].

It is known that sepsis orchestrates profound changes in the metabolic programs of both immune and non-immune (stromal and parenchymal) cells eventually leading to dysfunction of several organs such as the heart, lung, kidney, liver, and brain [15,16,17,18]. In sepsis, there is a metabolic shift in cellular energy generation pathways, similar to the phenomenon termed as Warburg effect observed in cancer cells [19], in which energy production is preferentially by glycolysis rather than the more efficient oxidative phosphorylation (OXPHOS) even in the presence of adequate oxygen levels [18,20,21]. The glycolytic switch that occurs during the initial stages of inflammation may be beneficial because it enhances the availability of metabolic intermediates to meet cellular biosynthetic and bioenergetic needs thereby promoting processes like cellular growth, differentiation, and effector function [22]. However, inability of the cellular metabolic machinery to restore OXPHOS and reinstate metabolic homeostasis at a later stage may result in organ dysfunction [23].

The pathophysiology of sepsis involves complex intercellular interactions, in which not only soluble mediators but also extracellular vesicles including exosomes play an important role. Exosomes are biological nanoparticles with a size range of 30–150 nm [24]. They are released by a plethora of cells and are capable of reprogramming response of local or distant target cells through delivery of bioactive molecules including proteins, nucleic acids (DNAs, messenger RNAs, microRNAs), and lipids. Thus, exosomes are recognized as important cargo particles encapsulating mediator payloads in the regulation of cellular communication [24]. Recent evidence indicates that exosomes significantly affect metabolic programs of their recipient cells through transfer of their payloads thereby regulating outcomes such as inflammation in sepsis [25] and metastasis in cancer [26]. In this review, we describe the metabolic changes occurring in endothelial cells and immune cells during sepsis, which is followed by the perspective about the potential role of exosomes in mediating cellular metabolic remodeling in sepsis.

Here, we have cited and discussed only some of those studies that are selected based on our current research interest. We thus have to express our sincere apology that many original and/or critical studies were not cited due to limited space of this review.

## 2. Metabolic Dysfunction and Regulation in Sepsis

### 2.1. Metabolic Regulation of Hyperinflammation

Innate immune cells, notably macrophages, neutrophils, and dendritic cells, constitute host frontline defense against invading pathogens and concurrently function as inducers of adaptive immunity, a pathogen-specific immune response, mediated by B and T cells. Innate immune cells express receptors known as pattern recognition receptors (PRRs) which recognize conserved microbial motifs referred to as pathogen-associated molecular patterns (PAMPs). Additionally, PRRs recognize damage-associated molecular patterns (DAMPs) released from damaged host cells. The best studied PRRs include the Toll-like receptors (TLRs), Nuclear-binding oligomerization domain (NOD)-like receptors (NLRs), Retinoic acid-inducible gene (RIG)-like receptors (RLRs), and the C-type lectin-like receptors (CLRs). Following infection, immune cells are activated through recognition of PAMPs or DAMPs by PRRs to initiate an inflammatory response. This represents a natural host defense response aimed at eliminating invading pathogens. However, aberrant activation of these innate immune cells, such as that observed in sepsis, leads to a hyperinflammatory state characterized by increased release of pro-inflammatory mediators [27,28].

Sepsis-induced hyperinflammation is associated with energy deficits, which prompts alterations in cellular metabolism. Thus, there is a shift from OXPHOS to glycolysis in macrophages, neutrophils, and DCs [29,30]. Extensive studies have unearthed some underlying mechanisms of this metabolic switch. Activation of macrophages and DCs following LPS and interferon gamma (IFN-γ) stimulation upregulates expression of inducible nitric oxide synthase (iNOS) which produces nitric oxide (NO), a reactive nitrogen species [31,32]. NO suppresses mitochondrial respiration through nitrosylation of electron transport chain proteins such as cytochrome C oxidase and Complex I [33,34], while concomitantly increasing glycolytic flux.

The mammalian target of rapamycin/hypoxia-inducible factor alpha (mTOR/HIF-1α) pathway also promotes the switch to glycolysis. Treatment of macrophages and DCs with LPS increases expression of the transcription factor HIF-1α [35,36], possibly by mTOR-dependent activation of HIF-1α which occurs through interaction of the Raptor component of mTOR with its signaling motif located in the N terminus of HIF-1α [37]. A surge in HIF-1α levels consequently upregulate genes encoding inflammatory mediators and glycolytic proteins such as glucose transporter 1 (GLUT1), 6-phosphofructo-2-kinase/fructose-2,6-bisphosphatase (PFKFB3), hexokinase (HK2), pyruvate kinase (PKM2), and lactate dehydrogenase (LDH) [25,38]. Additionally, the transcription factor Zinc fingers and homeoboxes 2 (Zhx2) is also upregulated in macrophages after LPS stimulation and binds to the promotor region of PFKFB3 to increase its expression thereby driving glycolysis [39]. The inflammasome NLRP3 also augments glycolysis in macrophages after exposure to LPS and amyloid β through release of IL-1β [40]. IL-1β binds to IL-1 receptor type 1 (IL1R1) in an autocrine manner and promotes the expression of PFKFB3 [40]. IL-1β is a metabolic hormone which facilitates glycolysis in rat ovarian cells [41], and its induction of glycolysis may also involve HIF-1α [38]. Adenosine monophosphate-activated protein kinase (AMPK) antagonizes glycolysis and is known to promote β-oxidation of fatty acids by upregulating intermediates such as peroxisome proliferator-activated receptor γ (PPAR-γ) and carnitine palmitoyl transferase 1 (CPT1). Consequently, LPS stimulation of macrophages and DCs downregulate AMPK thereby impairing OXPHOS and promoting glycolysis [18].

In neutrophils, increased glycolysis augments the formation of neutrophil extracellular traps (NETs) [30], by which neutrophils trap and eliminate invading pathogens [42]. Increased glycolysis in neutrophils may, however, inhibit their migration to sites of infection thereby perpetuating inflammation in sepsis due to limited bacterial clearance [43].

### 2.2. Metabolic Regulation of Immunosuppression

The induction of anti-inflammatory response aims at diminishing inflammation and initiating tissue repair. However, excessive inhibition of leukocytes, as observed in sepsis, may lead to immune paralysis. In this state, immune cells are unable to mount appropriate responses to inflammatory stimuli thereby making the host vulnerable to infections. Defects in cellular metabolic pathways underlie this feature. Cheng et al. observed defective glycolysis, β-fatty acid oxidation, and OXPHOS in monocytes rendered immunotolerant in vitro [44]. These metabolic defects were evidenced by decreased lactate production, downregulated expression of fatty acid transporters CD36 and CPT1, and decreased oxygen consumption [45]. Human leukocyte antigen-DR (HLA-DR) or major histocompatibility complex (MHC) class II molecule expression is key to the activation of adaptive immunity by antigen-presenting cells (APCs) such as dendritic cells, macrophages, and B cells [46,47]. Immunotolerant APCs, however, show repressed expression of HLA-DR, suggesting that the cellular metabolic defects contribute to immune paralysis in sepsis.

As mentioned earlier, lymphocyte apoptosis represents another cause of immunosuppression in sepsis and correlates with poor prognosis. PPAR-γ suppresses pro-inflammatory response in immune cells such as macrophages by fostering the β-fatty acid oxidation metabolic pathway [11]. PPAR-γ has been shown to induce T-cell apoptosis in both human and murine sepsis [48,49]. PPAR-γ induced T-cell apoptosis through inhibition of the PI3K/Akt signaling [48], which is associated with its downstream target, mTOR, that mediates glycolysis. Indeed, Akt signaling mitigates lymphocyte apoptosis and improves survival in septic mice [50]. Moreover, autophagy, a cytoprotective and energy-conserving cellular recycling process, induced by AMPK under nutrient poor conditions, exerts an influence on T cells. Accordingly, it has been shown that inhibition of autophagy contributes to T-cell apoptosis in sepsis, suppresses T cell effector functions, and increases mortality [51,52].

The immunosuppressive role of MDSCs in sepsis is well documented. Darcy et al. reported the suppression of T cell proliferation and function by arginase-expressing MDSCs, in which metabolism of the amino acid, L-arginine, by MDSCs suppressed expression of T cell zeta chain [53]. Recently, Ohl et al. have described a nuclear factor (erythroid-derived 2)-like (Nrf2) mediated expansion of MDSCs in sepsis. These MDSCs were highly immunosuppressive and increased T cell apoptosis in vitro [54]. Transcriptomic analysis identified upregulation of glycolytic and pentose phosphate pathway related genes in the MDSCs. Moreover, the MDSCs showed increased glucose uptake required for their generation in vitro, thereby showing elevated glycolysis following LPS stimulation [54].

Because the complexity of metabolic relationships between hyperinflammation and immunosuppression hinders the development of clinically effective therapeutics for sepsis [11], further investigations are required to identify metabolic and immunologic pathways and their molecular mechanisms by which host immune system is either impaired or rescued during sepsis.

## 3. Metabolic Reprogramming in Tissue Tolerance during Sepsis

**Figure 1:**
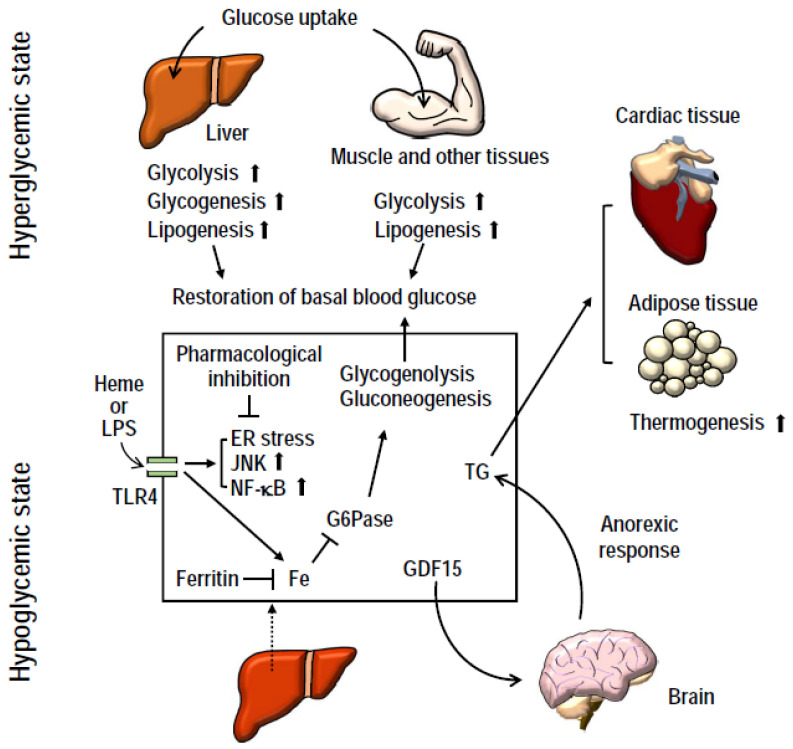
Glucose and triglyceride metabolism in the maintenance of tissue tolerance in sepsis. Maintaining normal blood glucose levels is important for survival in sepsis. To correct hyperglycemia in the early stages of sepsis, glucose uptake in liver and other tissues such as muscles is enhanced. At a later stage in sepsis, lethal hypoglycemia may develop. Tolerance mechanisms such as the sequestration of iron (Fe) ions by ferritin, and inhibition of ER stress and inflammatory pathways sustain G6Pase levels ensuring hepatic glucose production and output for peripheral utilization. Additionally, infection-induced anorexic response causes a switch in energy substrate from glucose to triglyceride. Endocrine communication via the brain–liver axis results in hepatic triglyceride production which maintains the function of cardiac and adipose tissue.

Tolerance to infection does not alter pathogen burden and inflammatory response; it improves host endurance and survival [55,56] and thus is emerging as a key determinant of sepsis survival. Maintenance of tissue tolerance may, therefore, be indispensable in the management of sepsis. To ensure survival amidst the numerous complications engendered by septic inflammation, adaptive responses through systemic metabolic changes have evolved to support tissue function and maintain normal physiological processes. Glucose and triglycerides are two important substrates utilized for cellular energy production. Their regulation is essential in establishing disease tolerance (Figure 1). Therefore, we discuss the impact of glucose and triglyceride metabolism on tissue tolerance in sepsis.

### 3.1. Glucose Metabolism

Deregulation in glucose metabolism correlates with sepsis severity. A hyperglycemic response, which results from pronounced insulin resistance and altered glycogen metabolism, is often observed at the early stages of sepsis. Induced hyperglycemia may be beneficial in that it ensures availability of glucose to cells to satisfy their immediate bioenergetic demands under inflammatory conditions where energy production through mitochondrial respiration is severely impaired [57]. In some instance in sepsis, hypoglycemia may also occur, which may derive from infection-associated anorexia, depletion of glycogen stores, glucose malabsorption, and increased peripheral glucose utilization [58,59,60]. 

Tolerance mechanisms that maintain a fine balance in glucose metabolism (glucose output and peripheral utilization) counteract these potentially lethal perturbations. One of such mechanisms is restoring insulin sensitivity and hepatic levels of the rate limiting gluconeogenic and glycogenolytic enzyme, glucose-6-phosphatase (G6Pase) [61]. Da Silva et al. show that drug-induced suppression of endoplasmic reticulum (ER) stress and Toll-like receptor (TLR) downstream signaling (including JNK and NF-κB activation) prevented both hyperglycemia and hypoglycemia in septic rats by improving insulin signaling and restoring hepatic G6Pase level, respectively [62]. These correlated with better disease tolerance and improved survival. Upregulation of ferritin, a hetero-polymeric protein, during septic insult has been shown to antagonize lethal hypoglycemia in polymicrobial sepsis by mitigating the suppression of G6Pase. This G6Pase suppression was mediated by either heme-TLR4 signaling or the generation of reactive radicals from heme following hemolysis. Ferritin executes this role by chelating free iron ions, and additionally oxidizing toxic divalent Fe^2+^ ion to the inert trivalent Fe^3+^ ion through its ferroxidase activity leading to maintenance of the minimum glucose levels required for generating tolerance [59]. Neutrophil gelatinase-associated lipocalin (NGAL) is an acute-phase molecule whose expression is increased during inflammation [63]. Upon infection, NGAL deprives bacteria of iron and thereby functions as a bactericidal protein due to its capability as a potent iron-chelator [64].

The adaptive response in glucose metabolism may, however, be pathogen-specific because recovery from the mild hypoglycemia induced by treatment with TLR3 agonist poly(I:C), in a model of viral sepsis was independent from G6Pase activity [59]. Indeed, glucose requirements in viral and bacterial sepsis have been noted to be different with glucose utilization in the host being protective in the former but detrimental in the latter. In viral inflammation, glucose supplementation and utilization maintain neuronal function and promotes survival through inhibition of type I IFN-induced ER stress, and subsequently prevents CHOP-mediated neuronal apoptosis. 

It is noteworthy to probe the mechanism by which viral sepsis affects host immune responses, of either hyperinflammation or immunosuppression. Intriguingly, the patients with severe coronavirus disease 2019 (COVID-19) exhibited a tendency to have both hyperinflammation (e.g., increased cytokines) and immunosuppression (e.g., lymphopenia) [65]. Therefore, characterization of immunologic and metabolic features of pathogenesis in patients with SARS-CoV-2-induced sepsis, compared to those with bacterial or protozoan sepsis as well as with nonseptic but severe COVID-19 remains to be examined.

Glucose supplementation in endotoxemic or polymicrobial sepsis mice impaired both glucose disposal and insulin sensitivity, and induced pancreatic insufficiency leading to hyperglycemia and death [66]. In a mouse model of protozoan infection-induced sepsis, inhibition of glycolysis conferred protection against development of cerebral malaria through decreased formation of microthrombi, RBC sequestration, and hemorrhagic lesions in the brain of *Plasmodium berghei* ANKA-infected mice [67]. Thus, different pathogen classes may elicit divergent cellular stress responses requiring specific metabolic programs to maintain tissue tolerance. Moreover, these animal studies and a large randomized control trial involving critically ill patients in the intensive care units (ICUs) [68] suggests that maintaining blood glucose within optimal ranges may be essential to much favorable outcomes in sepsis. 

**Figure 2:**
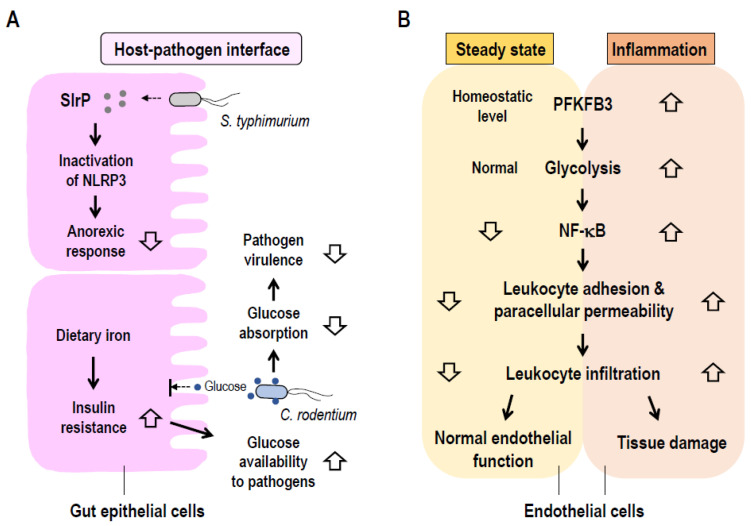
Metabolic regulation at host-pathogen interface and endothelial cells in sepsis. (**A**) Regulated anorexic response and infection tolerance may also emanate from interactions between host and pathogens. *S. typhimurium*-secreted Salmonella leucin-rich repeat protein (SlrP) involves in epithelial NLRP3 inflammasome inactivation to downregulate anorexic response in host. Host-pathogen metabolic programs ensure adequate supply of nutrients (glucose) to infectious pathogens (*C. rodentium*) at the interface, which result in attenuation of pathogen virulence. (**B**) Endothelial cells heavily rely on glycolysis, in which the glycolytic enzymes 6-phosphofructo-2-kinase/fructose-2,6-bisphosphatase isoform 3 (PFKFB3) plays the key role. In inflammation that causes aberrant activation of PFKFB3, increased glycolysis drives expression of inflammatory mediators, breakdown of the endothelial barrier, and increased extravasation of leukocytes into surrounding tissues. These pathologic events may lead to tissue damage. Upward and downward open arrows indicate increase and decrease, respectively.

*Salmonella typhimurium* (*S. typhimurium*) effector, Salmonella leucin-rich repeat protein (SlrP), inhibits anorexic response in its host through NOD-, LRR-and pyrin domain-containing protein 3 (NLRP3) inflammasome inactivation and mitigation of lamina propria myeloid cell-derived IL-1β signaling to the hypothalamus via the vagus nerve. The host and pathogens communicate to ensure availability of nutrients to the pathogen and culminate into attenuation of its virulence which protects the host [69]. In addition, iron-fed infected mice showed that iron indirectly suppressed virulence of *Citrobacter rodentium* (*C. rodentium*) by inducing systemic insulin resistance, which increased glucose availability to the pathogen through decreased absorption from the lumen of the intestines [70]. These models for host-pathogen communications at the interface for adaptive metabolic responses promoting host defense and survival are illustrated in Figure 2A.

### 3.2. Triglyceride Metabolism

Another pathway for energy production in cardiac and adipose tissues is the breakdown of triglycerides to release free fatty acids (FFAs) from which energy is derived through β-oxidation. This is especially necessary during infections where starvation responses are triggered due to sickness behaviors such as anorexia [71]. Triglycerides are required to confer protection against organ dysfunction, as fuel for brown adipose tissue thermogenesis for the timely exit from the hypometabolic-hypothermic state induced by energy trade-offs between immunity and other maintenance programs such as homeothermy during immune activation [72]. Defective hepatic triglyceride production in endotoxemic or *Escherichia coli*-infected mice lacking the MAP kinase regulatory protein, MKP1, causes endothelial damage, pronounced dysfunction of multiple organs, and increased mortality [73,74]. Aside from being efficient energy sources under septic conditions, triglyceride-rich lipids can dampen inflammatory response by sequestering LPS and facilitating its degradation in the liver [75]. Thus, hepatic triglyceride production may be an essential adaptive response for proper tissue function especially in the fasted state during sepsis [76,77,78]. Hence, perturbations in lipid metabolism in sepsis may likely be detrimental to tissue tolerance, and survival. To summarize current observations on glucose and triglyceride metabolism during sepsis: (i) metabolic programs that support tissue tolerance in sepsis are important and sufficient for survival regardless of pathogen burden, or degree of inflammation; (ii) maintaining metabolic substrates within homeostatic ranges is necessary for maintenance of disease or tissue tolerance in sepsis; (iii) specific septic insults require specific metabolic programs to ensure disease or tissue tolerance and survival.

### 3.3. Endothelial Cell Metabolism in Sepsis

Endothelial cells (ECs) are highly plastic and exhibit diverse phenotypes under both physiological and pathological conditions. Over a decade’s research has led to the discovery that metabolic programs are cardinal to phenotypic switch, and function of ECs. Although ECs also exhibit metabolic plasticity [79], glycolysis has been identified as the main source of energy generation through glycolytic breakdown of glucose to lactate [19]. This evolutionary adaptation facilitates angiogenesis especially in avascular and hypoxic regions, protects ECs against ROS (generated through mitochondrial respiration) damage while also making available to perivascular cells adequate oxygen to meet their metabolic needs [80]. Thus, basal level of glycolysis is maintained in quiescent ECs, and further upregulated in activated ECs.

A major regulator of glycolysis in ECs is the enzyme 6-phosphofructo-2-kinase/fructose-2,6-bisphosphatase isoform 3 (PFKFB3). Hence, alteration of endothelial PFKFB3 significantly impairs ECs function (Figure 2B). Accordingly, endothelial-specific PFKFB3 knockout mice show defective lactate-mediated M2 macrophage polarization and skeletal muscle regeneration following ischemic injury [81]. Strikingly, endothelial PFKFB3-driven glycolysis contributes substantially to tumor growth [82], vessel sprouting and pathological angiogenesis [83,84]. Of particular importance to sepsis, aberrant PFKFB3-directed EC glycolysis foments EC dysfunction, which is a major pathologic feature that drives MODS. Pharmacological inhibition with 3-(3-pyridinyl)-1-(4-pyridinyl)-2-propen-1-one (3PO) or endothelial-specific genetic ablation of PFKFB3 inactivates NF-κB signaling in ECs and as a consequence limits leukocyte infiltration through inhibition of ICAM-1 and VCAM-1 expression culminating in attenuation of LPS-induced acute lung injury [85]. In vitro, siRNA knockdown of PFKFB3 inactivates NF-κB signaling in EA.hy926 human endothelial cell line [86]. Consequently, TNF-α-induced cytokine and ICAM-1 protein expression are suppressed. This provides further evidence of the involvement of PFKFB3-mediated EC glycolysis in vascular inflammation [86]. Endothelial PFKFB3 may therefore be a viable metabolic node that can be targeted for the treatment of inflammatory diseases. In cancer, inhibition of endothelial PFKFB3 with optimum dose of 3PO, induced tumor vessel normalization accompanied by decreased metastasis and improved response to chemotherapy [82].

Sepsis-induced endothelial dysfunction associates with impaired outcomes for coagulation, permeability, and leukocyte diapedesis, and further leads to multiple organ failure [87]. The ECs are thus considered to be the player pivotal to trigger sepsis pathogenesis; hence, it is imperative to characterize molecular mechanisms by which endothelial dysfunction is elicited during sepsis. Undoubtedly, better understanding of sepsis-induced alterations in metabolic pathways of EC dysfunction is important for improving treatment of sepsis through EC-targeted therapeutics.

## 4. Exosome Involvement in Sepsis-Induced Metabolic Changes

Accumulated evidence has established the seminal roles of exosomes in intercellular communication. In sepsis, exosomal transfer of bioactive molecules (proteins, microRNAs, mRNAs, etc.) between cells has been reported to associate with variable and often contrasting consequences (protective or harmful) observed between different studies [88]. Thus, research interest on exosomes as diagnostic, prognostic, or therapeutic agents in sepsis has piqued over the past decade [89]. Alterations in metabolic programs underlie the pathologic features of sepsis. However, compared to other inflammatory diseases like cancer, studies on the role of exosomes in metabolic reprogramming in sepsis have only begun gaining momentum. In this section, we consider the biogenesis of exosomes and present a hypothetical association between exosomes and metabolic reprogramming in sepsis.

### 4.1. Exosome Biogenesis 

Exosomes are small membranous vesicles (often with a size range of 30–150 nm) produced through the endosomal pathway and shed into the extracellular milieu through fusion of multivesicular bodies/endosomes (MVBs/MVEs) with the plasma membrane of the releasing cell [90,91]. The formation and release of exosomes begin with cell membrane invaginations known as endosomes. Endosomes represent cellular compartments encapsulating various extracellular and cytosolic components. [90,92,93]. During the maturation process of endosomes from early to late endosomes, there is a concomitant formation of intraluminal vesicles (ILVs; later released as exosomes) within the lumen of the endosome. ILVs are formed by the inward budding and scission of cargo-rich microdomains of the limiting membrane of the early endosome culminating into the formation of MVEs. At this stage, depending on their composition, MVEs may undergo degradation through fusion with lysosomes or move towards the cytoplasmic side of the plasma membrane where they fuse to release exosomes into the extracellular environment [94,95,96].

A class of lipids, sphingolipid (including ceramide and sphingosine-1-phosphate), plays multiple roles in regulating cellular physiological and pathological pathways [97]. Emerging evidence demonstrates that sphingolipids and their generating enzymes (e.g., sphingomyelinases) alter biogenesis and the function of exosomes in response to membranous stress [98]. Ceramide-containing vesicles were shown to deteriorate sepsis, and functional blocking of ceramide revealed to mitigate this syndrome in the studies with a mouse model of sepsis [99]. Further examinations into sepsis-induced alterations of sphingolipid metabolism would be helpful for elucidating the underlying mechanisms by which exosome production is altered with sepsis.

### 4.2. Exosomal Cargo and Sepsis Metabolic Reprogramming 

Exosomal transfer of cargo between tumor and cells in its microenvironment have been shown to promote metastasis [17]. In a recent study probing the role of triggering receptor expressed in myeloid cells 2 (TREM2)-expressing Kupffer cells (KCs; liver resident macrophages) in regulating lipid dysmetabolism in non-alcoholic fatty liver disease (NAFLD), Hou et al. showed that loss of TREM2 triggered release of KC exosomes which induced mitochondrial dysfunction in hepatocytes through transfer of miRNAs and worsened sepsis mortality in an NAFLD mice model [100]. These studies, among others, bespeak the relevance of exosomes in cellular metabolism.

**Figure 3:**
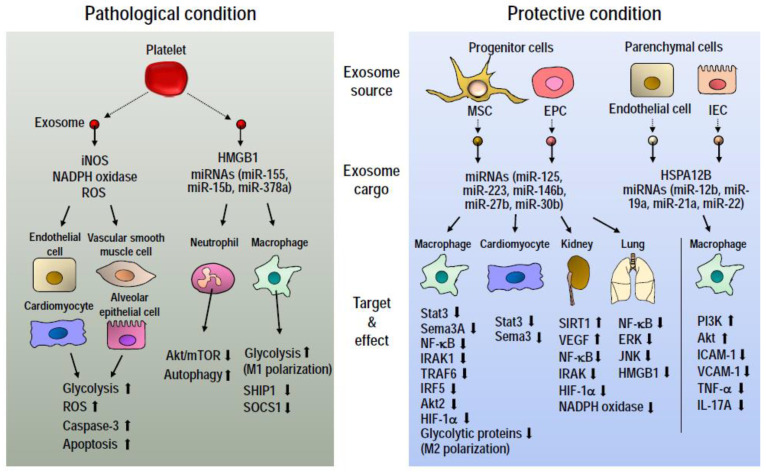
Potential roles of exosomes in the regulation of cellular metabolism in sepsis. Exosomes mediate both pathological and protective events in sepsis. Depending on their cargo/payloads and pathways modulated in target cells, metabolic roles of exosomes in sepsis pathophysiology may differ. During sepsis platelets release exosomes carrying bioactive molecules that promote inflammatory events and pathways associated with glycolysis in both parenchymal and immune cells. Exosomes originating from mesenchymal stem cells and endothelial progenitor cells (and in some cases, parenchymal cells) downregulate inflammatory pathways and preserve function of several tissues in sepsis; a disease phenotype akin to restoration of metabolic homeostasis. Upward and downward wide-tail arrows indicate increase and decrease, respectively.

Although direct evidence on exosome-mediated metabolic reprogramming remains sparse in the context of sepsis, we focus on the cargos of exosomes and their molecular targets and pathways. Exosomes modulate several signaling pathways, including the NF-κB, MAPK (JNK, p38, and ERK), and PI3K (Akt, mTOR) signaling pathways. These pathways are intricately linked to cellular metabolic programs [73,101,102,103]. Here, we discuss the molecular cargo, specifically proteins and miRNAs, of exosomes and the mechanisms by which they potentially reprogram cellular metabolic responses in sepsis (Figure 3).

### 4.3. Exosomal Proteins

Exosomes in sepsis have been demonstrated to contain enzymes such as iNOS and nicotinamide adenine dinucleotide phosphate (NADPH) oxidase. iNOS catalyzes the production of reactive nitrogen species (RNS), predominantly NO, from amino acid substrates such as arginine, citrulline, and glutamine [104,105], whereas NADPH oxidase-mediated electron transfer from NADPH to molecular oxygen provides an alternative pathway for superoxide generation [106]. Although moderate levels of RNS and ROS have been demonstrated to be essential for physiological processes, their accumulation, for instance under cellular stress conditions (e.g., septic inflammation), may impair mitochondrial respiration through the inhibition of respiratory complexes involved in the electron transport chain [33,34]. Under such conditions, mitochondrial dysfunction happens in tandem with a metabolic switch to the glycolytic pathway which may contribute to organ dysfunction when these metabolic anomalies are perpetuated.

In mice models of endotoxemia and polymicrobial sepsis, circulating exosomes showed the carriage of hydrogen peroxide transferable to cardiac endothelial cells both in vitro and in vivo where it induced the formation of podosome clusters, fragmentation of the tight junction protein, zonula occludens-1 (ZO-1), and consequently endothelial hyperpermeability [107]. Cardiac and endothelial dysfunction are associated with increased glycolysis in sepsis [21,85,86,108]. Again, increased glycolysis and ROS production induce apoptosis of alveolar epithelial cells in septic mice [109]. In cancer cells, ROS upregulates glycolysis, although this feature is largely an adaptive response to counteract ROS and augment survival [110]. The foregoing evidence suggests that exosomes may serve as biological agents capable of mediating redox and glucose metabolic alterations that eventually contribute to vascular, endothelial, and myocardial dysfunctions in sepsis. 

Ex-vivo culture of LPS-treated platelets released exosomes containing high mobility group box 1 (HMGB1) which caused the formation of neutrophil extracellular traps (NETs) in polymorphonuclear neutrophils (PMNs) through the repression of Akt/mTOR metabolic pathway and the induction of autophagy [111]. The ability of mesenteric lymph (ML) exosomes of gut epithelial cell origin obtained following trauma and hemorrhagic shock, to elicit pro-inflammatory response in alveolar macrophages, was partly dependent on the integrity of their surface proteins although the specific exosomal protein was not delineated. The ML exosomes promoted the M1 phenotypic switch by inducing NF-κB and iNOS expression in alveolar macrophages through TLR4 signaling leading to acute lung injury (ALI) [112]. Endothelial exosomes enriched in heat shock protein A12B (HSPA12B) mitigate pro-inflammatory responses in macrophages leading to the amelioration of cardiomyopathy in polymicrobial sepsis mice [113]. HSPA12B reportedly exerts this effect by upregulating the PI3K/Akt pathway [114].

### 4.4. Exosomal miRNAs

MicroRNAs (miRNAs) are important short single-stranded, noncoding RNA molecules that regulate gene expression by binding to the 3′-untranslated region of target mRNA to either inhibit translation or degrade the mRNA. miRNAs, together with other RNA species, are released in large quantities into circulation during sepsis and may modulate inflammatory response through known metabolic pathways as stated previously. Intriguingly, most of these miRNAs are packaged and transported via exosomes [102,115]. 

Exosomal miRNAs play heterogenous roles in disease pathogenesis by either fostering or mitigating pathological pathways potentially through the regulation of metabolic programs. In cancer, exosomes have been demonstrated to transfer miRNAs between tumor and stromal cells in the tumor microenvironment leading to the modulation of metastasis through alterations in key metabolic programs such as glycolysis, fatty acid oxidation, and OXPHOS [26,116]. In a pilot study, Real et al. identified altered levels in 30 and 65 exosome-associated miRNAs in ICU patients with septic shock on days 0 and 7 respectively after onset of septic shock. Through pathway analysis, mRNAs involved in IL-6, NF-κB, and PPAR signaling were observed among key targets of the differentially expressed miRNAs at both time points [117]. miR-15b-5p and miR-378a-3p contained in platelet-derived exosomes from septic patients could inhibit Akt/mTOR signaling by suppressing phosphoinositide-dependent protein kinase 1 (PDK1) leading to autophagy and NET formation in PMNs [111]. Serum exosomes expressing high levels of miR-155 following LPS-induced ALI could promote M1 polarization and pro-inflammatory responses in macrophages by suppressing Src homology 2 domain containing inositol polyphosphate 5-phosphatase 1 (SHIP1) and suppressor of cytokine signaling 1 (SOCS1) [118]. SOCS1 negatively regulates TLR and NF-κB signaling and thus its inhibition upregulates glycolysis and pro-inflammatory response in myeloid cells in septic mice through the STAT3/HIF-1α axis [119].

Exosomes may also modulate metabolic pathways to improve survival in sepsis. In this context, exosomes of mesenchymal stem cell (MSC) and endothelial progenitor cell (EPC) origins have been widely explored. Wang et al. shows that bone marrow MSC-derived exosomes confer protection against cardiac dysfunction in septic mice and attenuates systemic inflammatory response through exosomal miR-223-mediated suppression of inflammatory genes sema3A and stat3 in macrophages and cardiomyocytes [120]. Umbilical cord MSC-derived exosomes enriched in miR-146b ameliorated sepsis-related ALI [121] and dampened kidney injury in polymicrobial septic mice through the suppression of IL-1 receptor associated kinase (IRAK) and NF-κB signaling [122]. miR-27b contained in bone marrow MSC-derived exosomes maintained hepatic, renal, and pulmonary function in septic mice. Upon internalization by macrophages in vitro, the exosomes were demonstrated to transfer miR-27b that targets and downregulates the H3K27 demethylase, Jumonji D3 (JMJD3), thereby preventing the transcription of pro-inflammatory genes in synergy with NF-κB p65. This was revealed by the decreased enrichment of both transcription factors in the promoter regions of TNF-α, IL-1β, and IL-6 [123]. 

In vitro, bone marrow MSC-exosome-associated miR-30b-3p inhibited secretion of the acute phase reactant, serum amyloid A3 (SAA3) from type II alveolar epithelial cells. When administered intravenously in LPS-treated mice, exosomal miR-30b-3p downregulated phosphorylated forms of NF-κB p65, IκB, ERK, MEK1/2, p38, and JNK thus alleviating lung injury [124]. MSCs pretreated with inflammatory agonists have been shown to release exosomes superior in preserving organ function in sepsis. IL-1β-treated MSCs release exosomes that safeguard hepatic and pulmonary function by fostering an M1 to M2 macrophage transition through miR-146a-mediated suppression of key regulators of inflammation namely IRAK1, TRAF6, and IRF5 [125]. Similarly, exosomes from LPS-treated bone marrow MSCs downregulated NF-κB p65, Akt2 thereby promoting M2 polarization of macrophages. These exosomes decreased cardiomyocyte apoptosis and maintained cardiac function in a myocardial infarction mouse model, in vivo [126]. 

Zhou et al. report that exosomes derived from endothelial progenitor cells encapsulated elevated levels of miR-126-3p and miR-126-5p which targeted and suppressed HMGB1, VEGF-α, and phosphoinositide-3-kinase regulatory subunit 2 (PI3KR2) thereby salvaging lung function in animal models of endotoxemia and polymicrobial sepsis [127,128]. Indeed, it has been shown that endothelial HSPA12B upregulates miR-126 in circulating exosomes and that exosomal miR-126 suppresses expression of ICAM-1 and VCAM-1, as well as leukocyte infiltration into the myocardium of septic mice culminating in the maintenance of cardiac function [129]. Adipose tissue MSC-derived exosomes revealed to dampen septic inflammation and preserve kidney function by suppressing NF-κB p65, HIF-1α, and NADPH oxidase activity while upregulating SIRT1 and VEGF expressions [130,131]. In line with finding of regulatory function of exosomes, we have recently demonstrated the release of exosomes with altered miRNA composition into the intestinal lumen of septic mice [132]. These luminal exosomes showed a downregulation in messages of TNF-α and IL-17A in the inflamed intestinal tissues [132]. Hence, exosomes luminally released from gut epithelia are thought as regional mediators capable of dampening intestinal inflammation during sepsis potentially through delivery of their miRNAs. 

## 5. Conclusions

Recent advances have led to the understanding that flaws in metabolic programs in immune and parenchymal cells are instrumental in the pathophysiology of sepsis. These metabolic changes involve intertissue and/or intercellular communication through release of secretory factors such as cytokines, chemokines, growth factors, and exosomes. Exosomes are of particular importance since they are capable of packaging, preserving, and shuttling most of these bioactive molecules to target cells. How exosomal biomolecules modulate catabolic and anabolic processes, and the impact on inflammatory response and/or immunosuppression in immune and parenchymal cells leading to organ dysfunction in sepsis remains scanty. Further studies are required to elucidate the mechanistic basis of exosome-mediated metabolic remodeling in sepsis.

## Data Availability

Not applicable.
